# Quality of life in cutaneous leishmaniasis patients in Khyber Pakhtunkhwa, Pakistan

**DOI:** 10.12669/pjms.41.1.9186

**Published:** 2025-01

**Authors:** Shams ur Rehman, Muhammad Sohaib, Saima Aleem, Khalid Rehman

**Affiliations:** 1 Shams ur Rehman Health Department, Govt. of Khyber Pakhtunkhwa, Peshawar, Pakistan; 2 Muhammad Sohaib Institute of Public Health & Social Sciences, Khyber Medical University, Peshawar, Pakistan; 3 Saima Aleem Institute of Public Health & Social Sciences, Khyber Medical University, Peshawar, Pakistan; 4 Khalid Rehman Institute of Public Health & Social Sciences, Khyber Medical University, Peshawar, Pakistan

**Keywords:** Dermatology, Cutaneous Leishmania, Quality of Life, Dermatology Life Quality Index, DLQI, Social Stigma

## Abstract

**Objective:**

To determine the impact of leishmaniasis on the quality of life of patients visiting the district headquarter hospital in District Khyber, Khyber Pakhtunkhwa, Pakistan.

**Methods:**

A cross-sectional exploratory study was conducted in District Headquarter Hospital of Khyber District, Khyber Pakhtunkhwa from November 2019 to April 2020. In this study, 349 participants who consented were included by using a non-probability consecutive sampling technique. A validated questionnaire “Dermatology Life Quality Index” (DLQI) was used for data collection. Independent sample t-test and one-way ANOVA were used for mean scores comparison.

**Results:**

The mean DLQI score of cutaneous leishmania patients was 11.83 ± 3.94. Cutaneous leishmaniasis (CL) patients experienced a huge impact on their quality of life. The highest effect was observed for symptoms and feelings while the least impact was for the treatment. Statistically, no significant difference in mean DLQI score was found for gender, age, marital status, lesion activity, lesion appearance, and treatment history (*p > 0.05*). However, statistically, a significant difference was observed for the education, occupation, lesion location, and lesion type (*p < 0.05*).

**Conclusion:**

CL had a significant impact on the patient’s quality of life and mental health. Further research is required to assess the impact of the treatment on QOL in CL patients. and for a better understanding of the issue and its mechanistic basis.

## INTRODUCTION

Leishmaniasis is a vector-borne disease caused by the Leishmania parasites, a group of protozoan diseases from over 20 different species transmitted to mammals, including human beings, by a female phlebotomine sandfly and being a class I disease, it affects the population in both developed and lower-middle-income countries.^1^ The infection comprises a variety of clinical manifestations that include cutaneous leishmaniasis (CL), visceral leishmaniasis (VL), and mucocutaneous leishmaniasis (MCL) with different degrees of severity depending upon the type of species involved and the immune status of the patient or host.^2^ However, the major drivers contributing to the spread are diverse ranging from malnutrition, and weak immune systems to population displacement, environmental changes, poor sanitary conditions, and poor financial resources.^3^

Globally, Leishmaniasis is found in about 89 countries^4^ and is considered a serious public health problem in the Eastern Mediterranean Region (EMR) including Pakistan. Currently, 95% of these cases occur in the Americas, the Mediterranean Basin, the Middle East, and Central Asia. It is estimated that 600,000 to one million new cases occur worldwide annually.^5^

Cutaneous leishmaniasis (CL) is the most common form of leishmaniasis. In the last decade, the WHO EMRO region has seen the biggest outbreak of CL in the world.^6^ In Pakistan, CL is a major public health issue with CL cases being distributed in almost all provinces.^7^ In recent times, due to cross-border terrorism, and uncertainty across Afghanistan, CL emerged as an endemic disease in Khyber Pakhtunkhwa.

With advancements in health care research, quality of life (QoL) has emerged as an important aspect to be considered for the patient’s and individual’s well-being with a prime focus on the treatment modalities. Cutaneous leishmaniasis, like many other challenging issues, has a stigma associated with it in many communities.^8^ Despite being non-fatal, the social and psychological health of patients is impacted by lesions and scars leading to both anxiety and depression and impaired quality of life, especially for those residing in poverty-stricken areas.^9^

In Khyber Pakhtunkhwa, being an endemic province to CL in Pakistan, to the authors’ knowledge, no study to date has measured the impact of leishmaniasis on the QOL of people affected by the disease in the current study setting. For this reason, the concept of this current study was conceived to determine the impact of leishmaniasis on the QOL of affected people in Khyber District, Khyber Pakhtunkhwa Province, Pakistan.

## METHODS

We conducted a single-centered cross-sectional study in the District Headquarters Hospital, Khyber District, KPK Pakistan. The DHQ hospital is catering to the highest number of CL cases in the district. The study was conducted from November 2019 to April 2020.

### Ethical Approval:

The study was approved by the Ethical Review Board of Khyber Medical University, Peshawar (Ref No: KMU/IPHSS/ETHICS/2022/QO/0184, dated January 25, 2022).

### Sample

For this study, participants were recruited by using a non-probability consecutive sampling technique. Taking a 95% Confidence Interval and 5% margin of error, the calculated sample size using openepi software was 349.

### Inclusion & Exclusion Criteria:

The inclusion criteria for the study consisted of both male and female participants, aged between 16 and 70 years, who had been diagnosed with Leishmaniasis during the period from January 2019 to December 2019. Participants were excluded from the study if they were suffering from critical illnesses or had a history of chronic skin conditions such as scabies, as well as other chronic liver diseases. All the leishmania patients reported to the study setting were screened for inclusion criteria. Enrolled participants were informed about the study details, and written informed consent was taken validating volunteer participation.

### Questionnaire in Brief:

We gathered data by using a self-administered questionnaire comprising of two sections. The first section dealt with socio-demographic information and clinical aspects of the lesion including type, appearance, location, condition, and prior treatment history. The second section comprised of “Dermatology Life Quality Index” (DLQI) questionnaire” to assess the quality of life of study participants.

### Dermatology Life Quality Index:

Since 1994, both self-explanatory and self-administered, dermatology specific questionnaire in use is DLQI^10^, measuring impact of dermatological ailments on quality of life of the individuals. A total of ten questions in this tool are further grouped into six domains; symptoms and feelings (question one and two), daily activities (question three and four), leisure (question five and six), work and schooling (question seven), personal relationships (question eight and nine), and treatment domain (question 10) during the previous seven days.

The scores of each question range from 0-3. DLQI score was divided into five parts based on the acquired score: no effect (0-1), small effect (2-5), moderate effect (6-10), very large effect (11-20), extremely large effect (21-30) on patients’ life. The total DLQI score range from 0 (no effect) to 30 (greatest impairment)^10^,^11^.

### Data Analysis:

SPSS Version-26 was used for the data analysis. For continuous variables, data were expressed as mean and standard deviation. Quantitative variables were presented as frequencies and percentages. Independent sample t-test and one-way ANOVA were used to compare the mean. Statistical significance was based on *p <0.05*.

## RESULTS

### Socio-demographic Characteristics:

A total of 349 patients participated in this study having a mean age of 31.66 (± 11.03) years; males 33.8% and 66.2% females. All the baseline characteristics of study participants are summarized in Table-I.

**Table-I:** Baseline Socio-demographic details of Leishmania Patients.

Characteristics		Frequency	Percentage
Gender	Female	231	66.2%
	Male	118	33.8%
Age in years	16 - 30	190	54.4%
	31 - 50	142	40.7%
	51 - 70	17	4.9%
Marital Status	Single	43	12.3%
	Married	306	87.7%
Education	Below Diploma	292	83.7%
	Diploma	42	12.0%
	College	15	4.3%
Occupation	Unemployed	282	80.8%
	Employed	9	2.6%
	Self Employed	58	16.6%
Lesion Location	Head and Neck	73	20.9%
	Upper extremities	183	52.4%
	Lower extremities	93	26.6%
Lesion Type	Papular	182	52.1%
	Nodular	25	7.2%
	Plaque	142	40.7%
Lesion appearance	Ulcerative	13	3.7%
	Non-ulcerative	336	96.3%
Lesion activity	Active	346	99.1%
	Scar	3	.9%
Treatment history	Undertreatment	341	97.7%
	Without treatment	8	2.3%

### Quality of Life:

**Fig.1:**
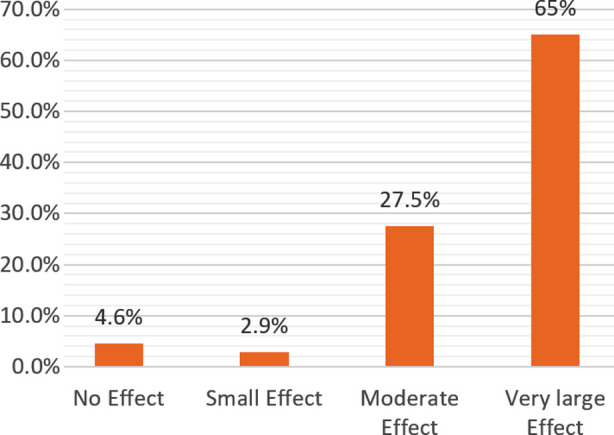
Impact of Leishmaniasis on Quality of Life of Study Participants.

Of the 349 patients, the location of the lesion in leishmania patients were mostly seen on upper extremities (52.4%), followed by lower extremities (26.6%), head and neck (20.9%), he lesions in 99.1% active s and 0.9% scars. Based upon the scores, a very large effect on the quality of life was experienced by 65% of study participants in comparison to 2.9% having a small effect and 4.6% having no effect on QOL associated to leishmania (Fig.1).

DLQI scores ranged from 0–30. A very large impact on the study participant’s quality of life as indicated by mean DLQI score of 11.83 (± 3.94). The highest impact (3.23 ± 1.11) was observed for the symptoms and feelings domain, while the lowest effect (0.002 ± 0.053) was observed for the treatment domain. Table-II projects the mean scores of DLQI’s domains.

**Table-II:** Leishmaniasis Patients DLQI Scores.

DLQI Domain	Mean ± S. D	Minimum	Maximum	Median	Interquartile range (IQR)
Symptoms and feelings	3.23 ± 1.11	0	6	4	2
Daily activities	2.16 ± .95	0	5	2	1
Leisure	1.81 ± 1.13	0	6	2	1
Work and school	1.68 ± 1.16	0	3	1	2
Personal relationships	2.93 ± 1.25	0	6	3	1
Treatment	.002 ± .053	0	1	0	0
Total scores	11.83 ± 3.94	0	20	12	3

### Dermatology Life Quality Index Score’s Association with Study Variables:

The association of both sociodemographic and clinical variables with the DLQI is presented in Table-III. There was no statistically significant difference (*p 0.144*) among the means DLQI scores in both males (12.26 ± 4.198) and females (11.61± 3.80) and with regards to age groups (*p 0.259*) and marital status (*p 0.271*). However, a significant difference in QOL was found for education (*p<.001*) with the maximum impairment was among the patients’ education with a diploma (13.97 **±** 2.66) and according to the patient occupation (*p <0.034*) with the highest impact on self-employed participants (13.05 **±** 3.78). Lesion’s location had significant effect on the quality of life of study participants (*p<0.003*) especially in patients having a lesion in the head and neck region (13.12**±** 2.61). Quality of life in patients with plaque lesions was better than those with nodular and papular (*p < 0.001*).

**Table-III:** Dermatology Life Quality Index Score’s Association with Study Variables.

DLQI Score			

Variables	N (%)	Mean ± SD	P-value
***Gender***			
Female	231 (66.2)	11.6104 ± 3.80214	.144
Male	118 (33.8)	12.2627 ± 4.19896
***Age in years***			
16 to 30 Years	190 (54.4)	11.7579 ± 4.13711	.259
31 to 50 Years	142 (40.7)	12.0915 ± 3.58069	
51 to 70 Years	17 (4.9)	4.58418 ± 4.58418
***Marital Status***			
Single	43 (12.3)	11.2093 ± 3.49529	.271
Married	306 (87.7)	11.9183 ± 4.00367
***Education***			
Below Diploma	292 (83.7)	11.5925 ± 3.90370	< 0.001
Diploma	42 (12)	13.9762 ± 2.66402	
College	15 (4.3)	10.4667 ± 5.71797
***Occupation***			
Unemployed	282 (80.8)	11.5993 ± 3.90807	.034
Employed	9 (2.6)	11.2222 ± 5.11805	
Self Employed	58 (16.6)	13.0517 ± 3.78094
***Lesion Location***			
Head and Neck	73 (20.9	13.1233 ± 2.61908	<0.003
Upper extremities	183 (52.4)	11.6940 ± 3.32396	
Lower extremities	93 (26.4)	11.0860 ± 5.45666
***Lesion Type***			
Papular	182 (52.1)	12.6593 ± 3.40854	< 0.001
Nodular	25 (7.2)	13.0000 ± 3.66288	
Plaque	142 (40.7)	10.5634 ± 4.30152
***Lesion appearance***			
Ulcerative	13 (3.7)	11.8462 ± 4.16025	.989
Non-ulcerative	336 (96.3)	11.8304 ± 3.94489
***Lesion activity***			
Active	346 (99.1)	11.8266 ± 3.95980	.825
Scar	3 (0.9)	12.3333 ± 2.30940
***Treatment history***			
Undertreatment	341 (97.7)	11.7889 ± 3.97226	.198
Without treatment	8 (2.3)	13.6250 ± 2.06588	

## DISCUSSION

In the current study, we studied the impact of cutaneous leishmaniasis on the quality of life of patients. The findings of our current study report a high impact on the quality of life of CL patients. The mean DLQI score of 11.83 ± 3.94 indicated a very large impairment of QOL. Cosmetic disfigurement, stigmatization, and lifestyle changes due to many skin diseases have been associated with psychological morbidity^12^,^13^ and lower quality of life. Likewise, cutaneous leishmaniasis is not a life-threatening disease, however, when the lesions are in cosmetically important areas including the face or on any exposed part of the body with scars, both self and social stigmatization emerge aggravating diverse psychological issues.^14^-^16^

The findings of our current study report a high impact on the quality of life of the CL patients. The mean DLQI score of 11.83 ± 3.94 indicated a very large impairment of QOL. Our findings were concordant with other studies reporting that cutaneous leishmaniasis (CL) impaired QOL and had a moderate to large negative effect on the QOL.^17^ Likewise, another study conducted in Turkey reported a moderate to high impact on the QOL of CL patients and impairment of psychological functioning resulting in depression and anxiety.^18^ The findings of our study were also consistent with that reported in a study conducted in Ethiopia where study participants presented the median DLQI score as 10 projecting the effect of CL on the quality of life of patients.^15^ The findings were also in agreement with those reported in a study conducted in Sri Lanka.^9^ The findings of a study conducted on the quality of life of post-kala-azar dermal leishmania reported the highest impact on symptoms and feelings domain (2.18 ± 1.08) which is similar to scores of the current study in the same domain (3.23 ± 1.11). Comparatively lowered self-esteem in CL patients negatively affecting their quality of life was reported in a study^8^ Other studies, too, have stressed the detrimental effect of CL on psychological and social aspects of life.^19^,^20^ The lowest score in the current study was for the treatment domain of DLQI (.002 ± .053), however, this was in contradiction to the findings of a study reporting a (2.06) score for the treatment domain.^10^ Based on the DLQI scores, the maximum influence in our study was on symptoms and feeling domain. This is in agreement with other similar studies.^16^,^21^

DLQI scores revealed that the quality of life of patients in the current study did not vary significantly for gender, age, marital status, or occupation, however, it was significantly associated with education (*p<0.001*), lesions location (*p<0.003*) and lesion type (*p< 0.001*). One possible explanation may be that the lesions appearing on more visible parts have more impact on the quality of life and overall mental health outcomes of the patients as reported by other studies.^17^,^21^

In Pakistan, the areas mostly affected are rural and remote. Leishmaniasis does not receive much importance when there are so many other challenges faced by the government and health departments. Communities in these rural and remote areas have low level of knowledge in regard to prevention and are highly vulnerable to social stigma due to the local social norms.

### Limitations:

The cross-sectional study design is a major limitation to establishing any causality and due to single single-centered study, the findings cannot be generalized.

## CONCLUSION

Based on the findings of the current study, the quality of life had significantly reduced in patients with cutaneous leishmaniasis particularly for symptoms, feelings, and personal relationship domain. The QOL in CL-affected patients was significantly associated with the education, occupation, type and location of lesion resulting in social and self-stigmatization and raising the risk of mental health issues.

### Recommendations:

Both preventive and curative activities should be increased to counter CL in the affected areas, especially during the transmission season. Counseling of patients should be done to minimize the psychological morbidity associated with CL.

### Authors Contribution:

**SR:** Concept development, data collection, data entry, data analysis, integrity of research.

**MS:** Tools development and validation.

**SA:** Literature search, writing of the manuscript and responsible for the accuracy of the study.

**KR:** Concept development, proofreading, and final approval of manuscript.

All authors have read the final version and are accountable for the integrity of the study.
